# Inflammatory proteins related to depression in multiple sclerosis: A systematic review and meta-analysis

**DOI:** 10.1016/j.bbih.2024.100939

**Published:** 2024-12-28

**Authors:** L.A. Kiropoulos, V. Rozenblat, N. Baes

**Affiliations:** Mood and Anxiety Disorders Lab, Melbourne School of Psychological Sciences, University of Melbourne, Victoria, Australia

**Keywords:** Inflammatory proteins, Depression, Multiple sclerosis, Meta-analysis

## Abstract

**Background:**

Up to 50% of individuals with multiple sclerosis (MS) experience depression. Depression has been accompanied by increases in inflammatory proteins. This meta-analysis summarized the data on inflammatory protein concentrations and level of depression in individuals with MS.

**Methods:**

We performed a meta-analysis of studies measuring inflammatory protein concentrations and level of depression in individuals with MS with a database search of the English literature (to October 2024) and a manual search of references. Quality of evidence was also assessed.

**Results:**

Fifteen studies involving measurements of inflammatory proteins and level of depression in 1102 individuals with MS were included in the meta-analysis: five for interleukin (IL)-10 (LPS and PHA), four for tumour necrosis factor (TNF)-α, four for interferon (IFN)-γ, and four for IL-6 (LPS and PHA). A meta-analysis showed that higher concentrations of TNF-α, IFN-γ, IL-6 and IL-10 were significantly associated with higher levels of depression in individuals with MS (*r* = 0.35, 95% CI [0.6,0.03], *p* = .015. Meta-analyses undertaken for individual inflammatory proteins of IFN-γ and IL-10 found positive associations between these proteins and level of depression although these did not reach statistical significance. Most studies were rated ‘poor quality’.

**Conclusion:**

This meta-analysis reports significant associations between higher concentrations of TNF-α, IFN-γ, IL-6 and IL-10 and level of depresson in individuals with MS. Future longitudinal studies with improved reporting of data are needed to replicate these results and confirm the mechanisms through which these inflammatory proteins are present. Meta-analytic findings lend support to depression being associated with the activation of the inflammatory system in individuals with MS.

## Highlights

• Up to 50% of individuals with MS experience depression.
• Depression has been accompanied by increases in inflammatory proteins.
• Meta-analysis showed association between TNF-a, IFN-g, IL-6 and IL-10 and depression in individuals with MS.
• Findings support activation of inflammatory system in individuals with MS and depression.
• Longitudinal studies with improved study design and reporting of data needed to replicate results.

## 1 Introduction

Multiple sclerosis (MS) is an immune-mediated chronic inflammatory and neurodegenerative disease of the central nervous system and is characterized by heterogeneity in illness course, clinical expression, and treatment efficacy (Browne et al., 2014; Ghielen et al., 2019; Polman, 2000). Common symptoms include fatigue, numbness, weakness and visual problems. It is the most common disease of the central nervous system in young adults with a ratio of 2 men to 3 women affected (Romeo et al., 2023). Higher levels of depression have been found in the first two years of an MS diagnosis (Janssens et al., 2003; Kiropoulos et al., 2016; Marrie et al., 2015; Tan-Kristanto and Kiropoulos, 2015). Point prevalence rates of depression ranging from 10 to 42% and up to 50% of individuals with MS experiencing depression in their lifetime (Arnett et al., 2008; Byatt et al., 2011) which is higher than in any other neurological illness (Suh et al., 2010) and the general population (Kruijshaar et al., 2005; Luppa et al., 2012).

There has been an interest in the role that inflammatory proteins and inflammation may play in the development of depression in those with MS (Arnett et al., 2008; Maes, 2008), however, there are no established inflammatory proteins of depression in MS and limited information on the role that inflammatory proteins may play in the pathogenesis of depression in MS. The ‘cytokine or inflammatory hypothesis’ (Maes, 2008) has been proposed to explain the development of depression in MS and states that elevated circulating levels of pro-inflammatory proteins might promote the evolution and maintenance of depressive symptoms (Maes, 2008).

There is much debate surrounding whether depression in MS is ‘organic’ due to increased inflammatory activity or ‘reactive’ due to a functional reaction to neurological and physical symptoms in MS (Rossi et al., 2017) or a combination of both of these factors (Rossi et al., 2017). The focus on the immune system and inflammatory proteins in the development of depression in MS is of importance and is supported by several findings. First, depression has been associated with pro- and anti-inflammatory proteins (Miller et al., 2009). Pro-inflammatory proteins identified include TNF-α, IL-6, IL-1β and C-reactive protein (CRP) which have been found to be increased in individuals with depression (Haapakoski et al., 2015; Miller et al., 2009). Certain anti-inflammatory proteins, such as IL-10, have also been found to be decreased in individuals with depression (Himmerich et al., 2019). A meta-analysis found higher concentrations of pro-inflammatory proteins TNF-a and IL-6 in individuals who were depressed compared to control subjects (Dowlati et al., 2010). Proteins have been shown to access the brain and interact with pathophysiological domains relevant to depression, including neurotransmitter metabolism, neuroendocrine function, and neural plasticity (Miller et al., 2009). Pro-inflammatory proteins, such as TNF-α, IL-1 and IL-6, are potent activators of the hypothalamic–pituitary–adrenal axis (HPA) and release of these proteins leads to hypersecretion of adrenal glucocorticoids which have been linked to depression. Increased concentrations of pro-inflammatory proteins are also thought to induce depressive symptoms by reducing the release of serotonin at synapses and as a consequence might also lead to the malfunctioning of noradrenergic and serotonergic circuits that represent pathways targeted by several anti-depressant drugs. Relatedly, the effects of these proteins are normalized following antidepressant treatment (Raison et al., 2006) suggesting that these proteins may be useful biomarkers of depressive episodes and treatment response, and influence the pathophysiology of depression (Raison et al., 2006).

Secondly, clinical depression may share a common pathological pathway, namely increased pro-inflammatory proteins, with MS given that MS also includes symptoms such as fatigue, reduced activity, social withdrawal and altered sleep patterns. This is reinforced by the finding that high rates of depression are found in neurodegenerative diseases such as MS which are associated with activation of inflammatory pathways (Maes, 2008). Relatedly, administration of IFN-γ has been found to be associated with ‘depressive’ and anxiety symptoms after 1–3 months of therapy and these symptoms may persist (Gold and Irwin, 2009). Separate research into MS and depression has suggested both to be accompanied by immune dysregulation (Rodríguez Murúa et al., 2022; Dowlati et al., 2010), specifically activation of inflammatory proteins which act as signalling proteins central for the immune responses and central to the pathogenesis of both MS (Martins et al., 2011) and depression (Dowlati et al., 2010).

Inflammatory proteins have also been identified as playing a major role in modulating the immune system, with pro- and anti-inflammatory proteins believed to play a prominent role in modulating the autoimmune inflammatory process in MS. These inflammatory protein levels have also been found to correlate with changes in MS activity. For example, inflammatory proteins have been found to be present in MS lesions, with IL-17 being one of the inflammatory proteins secreted mainly by activated T cells. Higher levels of IL-17 and IFN-γ in serum in individuals with relapse remitting MS (RRMS) and a negative correlation between level of ambulation and IL-17 and IFN-γ levels have also been found (Pasquali et al., 2015). Additionally, pro-inflammatory proteins such as IFN-γ, IL-2, IL-1β and TNF-α and anti-inflammatory proteins IL-4, IL-1 and IL-13 have shown to be significantly elevated in adults with MS compared to a healthy age and gender matched group (Martins et al., 2011).

Although associations between various proteins and measures of depression have been documented in individual studies in MS populations, there has been no pattern of inflammatory proteins related to depression defined in MS. No study has systematically examined the available results using meta-analytical techniques to examine the strength of evidence between inflammatory protein concentrations and level of depression in individuals with MS. Inflammatory proteins may serve as potential biomarkers that may assist in diagnosis, monitoring depression severity and treatment efficacy and determining prognosis of depression in MS. To address this gap in the literature, the aim of the current study was to undertake a systematic review and meta-analysis on the available literature to determine whether concentrations of inflammatory proteins were associated with level of depression in individuals with MS.

## 2 Methods and materials

Studies were included if they were original, published in English and included level of protein concentrations, level of depression or a diagnosis of Major Depressive Episode or Disorder based on DSM-IV criteria in individuals with MS. Records retrieved from searches conducted (earliest article to October 2024) of four databases (Embase, MEDLINE (Ovid), ProQuest and PsycINFO) were checked for duplicates and researchers (LK, NB, VR) independently reviewed titles and abstracts and full text studies of included studies. The search also included the ‘grey’ literature which included conference abstracts and student theses. In addition, LK and VR manually searched through the reference list of all studies to identify any additional relevant papers. Inconsistencies were discussed until consensus was reached with all authors.

The PRISMA (Preferred Reporting Items for Systematic Reviews and Meta-Analyses) guidelines were used ) and the study was registered with the International Prospective Register of Systematic Reviews (PROSPERO: CRD42020158215). Search terms were chosen following discussion with a medical librarian and the first author (LK) and these included terms with Boolean operators and search limits: “multiple sclerosis”, “depression”, “depressive” and “cytokine∗” or “interleukin∗” or “tumor necrosis factor∗” or “tnf” or “c reactive protein∗” or “crp” and “neuroinflamm∗” or “inflamm∗” in the article titles and abstracts of the respective databases.

The following information from the included full-text studies was extracted: 1) study characteristics (including study design, number of participants); 2) sample characteristics (including age, gender, time since MS diagnosis and MS type); 3) outcome variables (depression measure, inflammatory protein type and concentration, mean and standard deviation of level of depression); 4) psychological intervention for depression if relevant. Level of depression refers to participants’ total score on the self-report depression measure. Bipolar disorder was not part of the exclusion criteria and the search found no studies which examined level of depression and inflammatory proteins in individuals with bipolar disorder and MS. There were no restrictions on the type of media used from which the inflammatory proteins were quantified. Three contact attempts were made over a 3-month period with corresponding authors of manuscripts regarding missing data (n = 5).

Where data was available from three or more studies, random effects models were employed to estimate the summary effect size between level of depression and inflammatory protein concentration in MS samples. Between-study variance (τ2) was estimated using a restricted maximum likelihood method. All associations (positive and negative) and unadjusted coefficients were used in all meta-analyses. See Supplementary Table 1 for data checking procedures including transformation of data, examination of outliers, small study bias and heterogeneity. Prior to meta-analytic synthesis, correlation coefficients were transformed into the normally distributed Fischer's z for variance stabilization (Page et al., 2020) and reverted to Pearson's r for reporting the weighted average summary effect size (Andlauer et al., 2019 ).

A meta-analysis using grouped inflammatory proteins found in serum was undertaken followed by meta-analyses examining individual inflammatory proteins where at least three studies containing inflammatory proteins from serum were available. When data permitted, moderator analyses were undertaken to identify sources of heterogeneity for variables collected during the data extraction phase. Potential confounds of the relationship between inflammatory protein concentration and level of depression included presence of controls, study quality, type of assay, depression measure, study year, mean age of sample, percentage female, presence of disease modifying medication and years since MS diagnosis were investigated. The “metafor” (Viechtbauer, 2010) package for *R* (R Core Team, 2016) was used to perform meta-analytic calculations and data visualizations. Risk of bias in most identified studies was assessed using an adapted version of the Newcastle-Ottowa Quality Assessment Form for Cohort Studies. The methodological quality of Rahimlou et al. (2019) (Rahimlou et al., 2020) was assessed using the Cochrane Risk of Bias Tool for Randomised Control Trials.

## 3 Results

**Fig. 1:**
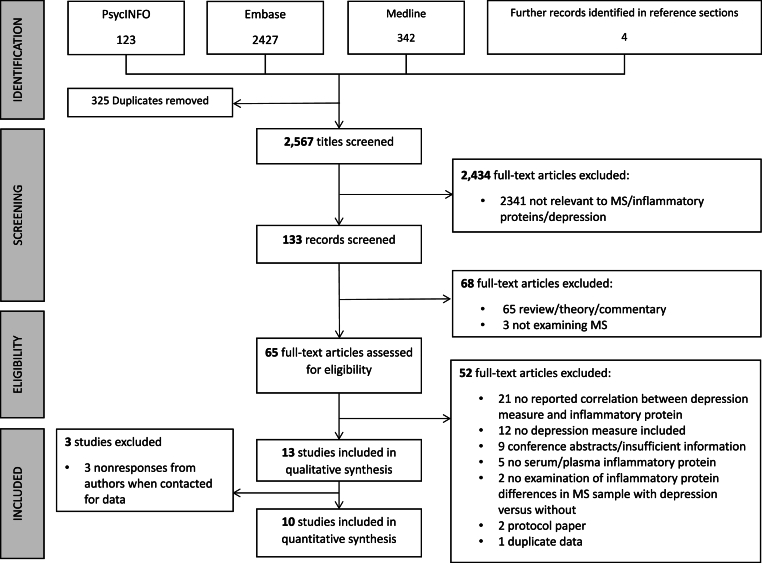
PRISMA flowchart of studies.

A total of 2894 records were identified from four databases (PsycINFO: 123; Embase: 2426; Medline: 341; Reference sections: 4) from the initial search for review. After removing duplicates, 2481 titles were screened for eligibility with 65 full text articles assessed for eligibility. Fifty two studies were excluded because they did not meet inclusion criteria due to not reporting an association between depression measure and inflammatory protein concentration (n = 21), no depression measure (n = 12), conference abstract or study with insufficient information (n = 9), no serum/plasma inflammatory protein (n = 5), protocol paper (n = 2), no examination of inflammatory protein in depressed and non-depressed samples (n = 1) and duplicate data (n = 1). Studies were also excluded because data was not provided by researchers even upon repeated request (n = 3), inflammatory protein effect sizes were provided in under three papers as meta-analyzing two data points is statistically inappropriate (n = 2), effect sizes for inflammatory protein related analyses were unavailable (n = 1). Odds ratios were able to be extracted for one study where correlations were not reported (Andlauer et al., 2019). Supplementary Table 2 lists the excluded studies. Supplementary Tables 3 and 4 list the inflammatory study characteristics. Fig. 1 displays the PRISMA flowchart (Page et al., 2020). Data and code for all analyses are available at the following link: https://osf.io/syrgk/?view_only=6663f8b55f6d411b856c81905ed8101b.

### 3.1 Types of inflammatory proteins related to depression in individuals with MS

In total, thirteen studies satisfied inclusion criteria with a total of 922 participants with MS. Table 1 displays the study characteristics for all thirteen studies included in this review: six studies for TNF-a (Andlauer et al., 2019; Heesen et al., 2005; Kahl et al., 2002; Patanella et al., 2010; Rolf et al., 2017; Rossi et al., 2014), six studies for IL-6 (Rahimlou et al., 2020; Patanella et al., 2010; Brenner et al., 2018; Ibrahim and Afifi, 2012; Koutsouraki et al., 2011; Sorenson et al., 2011), six studies for IL-10 (Heesen et al., 2005; Kahl et al., 2002; Patanella et al., 2010; Rolf et al., 2017; Sorenson et al., 2011; Mohr et al., 2001), four studies for IFN-γ (Kahl et al., 2002; Rolf et al., 2017; Sorenson et al., 2011; Mohr et al., 2001), three studies for CRP (Andlauer et al., 2019; Vesic et al., 2018), two studies for IL-4, (Kahl et al., 2002)^,^ (Mohr et al., 2001) two studies for sIL-6R (Ibrahim and Afifi, 2012; Koutsouraki et al., 2011), IL-8 (Rossi et al., 2017; Brenner et al., 2018) and one study for IL-1b (Rossi et al., 2017), IL-2 (Rossi et al., 2017) and IL-12 (Sorenson et al., 2011). Eight studies assessed inflammatory protein concentrations in serum (Rahimlou et al., 2020; Heesen et al., 2005; Kahl et al., 2002; Patanella et al., 2010; Rolf et al., 2017; Sorenson et al., 2011; Mohr et al., 2001; Vesic et al., 2018), and two studies assessed inflammatory protein concentrations in cerebrospinal fluid (CSF) (Rossi et al., 2014; Brenner et al., 2018). Eight studies reported inflammatory protein concentrations in pg/mL (Rahimlou et al., 2020; Kahl et al., 2002; Patanella et al., 2010; Brenner et al., 2018; Ibrahim and Afifi, 2012; Koutsouraki et al., 2011; Mohr et al., 2001; Vesic et al., 2018) and two studies in mg or ng/L. (Rahimlou et al., 2020; Vesic et al., 2018)

**Table 1:** Characteristics of included studies examining cytokine concentrations in MS samples with depression.

Study/Year	Cytokine measured	*N* (% female) (D, ND)	*M (SD)* Age (D, ND)	Depression measure
Andlauer et al. (2019)	TNF-α, IL-9, CRP	25 (88)/30 (77)	31.8 (NR)/32.1 (NR)	BDI-II
Brenner et al. (2018)	IL-6, IL-8	47 (31)/NR (NR)^a^	34ˆ^a^	MADRS-S
Heesen et al. (2005)	IFN-y, IL-10, TNF-a	23 (78%)/25 (80)	40.13 (2.23)^a^	ADS-L
Ibrahim and Afifi (2012)	IL-6	22 (NR)/12 (NR)	31.2 (8.7)^a^	BDI
Kahl et al. (2002)	TNF-α, IFN-y, IL-4, IL-10	16 (75)^a^	30.1 (5.8)^a^	BDI
Koutsouraki et al. (2011)	IL-6	13 (NR)/15 (NR)	34.62 (8.16)/38.2 (8.75)	BDI
Mohr et al. (2001)	IFN-y, IL-4, IL-10	14 (71)^a^	47.4 [29–69]^a^	BDI
Patanella et al. (2010)	TNF-α, IL-6, IL-10	30 (57)^a^	34.6 (5.7)^a^	BDI
Rahimlou et al. (2020)	IL-6	65 (72)^a^	42.15 (11.98)/39.9 (8.76)^b^	BDI-II
Rolf et al. (2017)	TNF-α, IFN-y, IL-10	40 (65)^a^	38.5 (7.8)/37.6 (9.6)^b^	HADS-D
Rossi et al. (2017)	TNF-α, IL-1b, IL-2, IL-8	405 (NR)^a^	NR (NR)/NR (NR)	BDI-II
Sorenson et al. (2011)	IFN-y, IL-6, IL-10, IL-12	42 (71.4)^a^	49 (9.11)^a^	POMS-TD
Vesic et al. (2018)	CRP	98 (66)^a^	41.7 (9.13)/38.6 (7.88)^c^	BDI

*Note.* ADS-L, Allgemeine Depressions-Skala (ADS); BDI, Beck Depression Inventory; BDI-II, Beck Depression Inventory, Version 2; POMS-TD.

Profile of Mood States-Total Mood Disturbance Score; HADS-D, Hospital Anxiety and Depression Scale – Depression; MADRS-S, Montgomery-Asberg Depression Rating Scale (self-rating version).

All inflammatory proteins were analyzed from serum samples except for Brenner et al., 2018 and Rossi et al., 2017 who reported inflammatory proteins taken from cerebrospinal fluid. Andlauer et al., 2019 reported inflammatory proteins taken from serum and cerebrospinal fluid for TNF-a, IL-9 and CRP.

a MS group totals as D/ND data was unavailable.

b MS Intervention/MS Control.

c RRMS/MS acute. Statistics exclude healthy control/without MS groups.

### 3.2 Associations between inflammatory concentrations and level of depression

Ten studies reported associations between inflammatory protein concentration and level of depression in participants with MS (Table 2; total N = 889; range: 14–405). Mean age of participants with MS was 44 (10.8) years (range: 30.1–49 years). Most participants were diagnosed with RRMS (710/79.8%) and seven studies included patients that were actively using disease modifying treatments (770/889; 86.6%) (see Table 2). Seven of the ten studies identified in Table 2 reported a significant correlation between inflammatory protein concentration and level of depression in MS participants: two studies for TNF-α with one using serum (Kahl et al., 2002) and the other CSF (Rossi et al., 2017), one study for IL-1β in CSF (Rossi et al., 2017), two studies for IL-6 with one using serum (Sorenson et al., 2011) and the other CSF (Brenner et al., 2018), two studies for IFN-γ in serum (Kahl et al., 2002; Mohr et al., 2001), one study for IL-10^31^, IL-10LPS and IL-10PHA (Sorenson et al., 2011) and CRP in serum (Vesic et al., 2018).

**Table 2:** Study characteristics and associations between cytokine concentrations and level of depression in individuals with MS.

Study/Year	Cytokine measured	N (%female)	MS Type	Age M (SD)	Depression Measure	*r* (p value) unadjusted	*r* (p value) adjusted	Variables controlled
Brenner et al. (2018)	Il-6 (CSF)	47 (31%)^c^	RRMS (*n* = 47)^b^	34^a^ (NR)	MADRS-S	0.41 (<0.01)	0.38 (<0.01)	Age, sex, EDSS score
	IL-8 (CSF)					−0.09 (>0.05)	−0.09 (>0.05)	
Heesen et al. (2005)	IL-10 (serum)	23 (78%)^c^	RRMS (*n* = 19)^b^ SPMS (*n* = 3) RPMS (*n* = 1)	40.13 (2.23)	ADS-L	0.59 (0.003)	NR	NR
Kahl et al. (2002)	TNF-α (serum)	16 (75%)NR	RRMS (*n* = 16)^b^	30.1 (NR)	BDI	0.55 (0.03)	NR	NR
	IFN-y (serum)					0.54 (0.03)	NR	
	IL-4 (serum)					0.13 (0.96)	NR	
	IL-10 (serum)					−0.01 (0.97)	NR	
Mohr et al. (2001)	IFN-y (serum)	14 (71%)^c^	RRMS (*n* = 14)^d^	47.4 (29.69)	BDI	0.56 (0.04)	NR	NR
	IFN-y + IL-10 (serum)					NR (0.04)	NR	
	IL-4 (serum)					NR (0.22)	NR	
Patanella et al. (2010)	TNF-α (serum)	30 (57%)^c^	RRMS (*n* = 30)^b^	34.6 (5.6)	BDI	0.09 (0.65)	NR	NR
	IL-6 (serum)					−0.12 (0.54)	NR	
	IL-10 (serum)					−0.02 (0.90)	NR	
	BDNF (serum)					−0.10 (0.58)	NR	
Rahimlou et al. (2020)	IL-6 (serum)	65 (72%)NR	RRMS (*n* = 65)^c^	42.15 (11.98)	BDI-II	NR	0.005 (0.98)	Age, sex, calorie intake
	BDNF (serum)					NR	0.36 (0.042)	
	NGF (serum)					NR	0.22 (0.23)	
Rolf et al. (2017)	TNF-α (serum)	40 (65%)NR	RRMS (*n* = 40)^b^	38.5 (7.8)	HADS-D	NR (>0.05)	NR	NR
	IL-10 (serum)					NR (>0.05)	NR	
Rossi et al. (2017)	TNF-α (CSF)	405 (NR)^c^	Total RRMS (*n* = 405)^b^ MS Acute (*n* = 54) Remission (*n* = 57)	NR (NR)	BDI-II	0.50 (<0.001)	NR	NR
	IL-1b (CSF)					0.52 (<0.001)	NR	
	IL-2 (CSF)					−0.06 (0.52)	NR	
	IL-8 (CSF)					0.11 (0.22)	NR	
Sorenson et al. (2011)	IL-6-LPS (serum)	42 (71.4%)NR	RRMS (*n* = 24)^d^ Progressive (*n* = 7) Unidentifiable (*n* = 9)	49 (9.11)	POMS-TD	0.02 (>0.05)	NR	NR
	IL-6-PHA (serum)					0.386 (<0.05)	NR	
	IL-10-LPS (serum)					0.435 (<0.01)	NR	
	IL-10-PHA (serum)					0.345 (<0.05)	NR	
	IFN-y-PHA (serum)					0.081 (>0.05)	NR	
Vesic et al. (2018)	CRP (serum)	98 (66%)^c^	RRMS (*n =* 50)^b^ MS acute (*n* = 48)	41.7 (9.13)	BDI	NR	0.40 (<0.001)	Sex, age, MS type, MS therapy, EDSS score

Note.

*M* = Mean. *SD* = Standard Deviation. ADS-L = Allgemeine Depressions-Skala Lang. MADRS-S = Montgomery-Asberg Depression Rating Scale, self-report. *r* = correlation. All MS participants listed in table had the RRMS type. All cytokines analyzed from serum samples except Brenner et al., 2018 and Rossi et al., 2017 who extracted inflammatory proteins from cerebrospinal fluid. NR = Not recorded. a = study included in meta-analyses.

NR = study did not report whether participants were taking anti-depressant medication and/or disease modifying treatment for MS.

a = Median value.

b = participants were on disease modifying medications for MS.

c participants were not taking anti-depressant medications.

d participants were not taking disease modifying medication for MS.

### 3.3 Differences in inflammatory protein concentrations in individuals with MS with and without depression

Three studies reported significant differences in inflammatory protein concentrations between MS participants with and without depression for IL-6 in serum (Ibrahim and Afifi, 2012; Koutsouraki et al., 2011), and IL-9,^30^ TNF-α (Andlauer et al., 2019) and CRP (Andlauer et al., 2019) in both serum and CSF (see Table 3). Of the data available, two studies found higher inflammatory protein concentrations in individuals with MS and depression for IL-6 (Ibrahim and Afifi, 2012; Koutsouraki et al., 2011) and sIL6R (Koutsouraki et al., 2011). Of the total 117 participants with MS in these studies, 51% (n = 60) were classified as depressed and 49% (n = 57) as non-depressed. Mean age of participants who were depressed was 32.54 (8.43) years compared to 33.8 (8.72) years who were not depressed.

**Table 3:** Studies examining cytokine concentrations in individuals with multiple sclerosis with and without depression.

Study	Cytokine measured	Gender (% female) (D, ND)	MS type (D, ND)	Age (yrs) M(SD) (D, ND)	Depression measure	Cytokine concentration (D, ND)	Mean difference in cytokine between D, ND (OR)
Andlauer et al. (2019)	CRP (CSF)	25(88)/30(77)	RRMS, SPMS, PPMS, CIS	31.8/32.1	BDI-II	NR/NR	OR=>1.53; p < .05
	CRP (serum)					NR/NR	OR=>1.53; p < .05
	IL-9 (CSF)					NR/NR	OR=>1.53; p < .05
	IL-9 (serum)					NR/NR	OR=>2.29; p < .05
	TNF-a (CSF)					NR/NR	OR=>1.17; p < .05
	TNF-a (serum)					NR/NR	OR=>2.07; p < .05
Koutsouraki et al. (2011)	IL-6 pg/mL (serum)	13(NR)/15(NR)	RRMS, SPMS	34.62 (8.16)/38.2 (8.75)	BDI	6.04(8.80)/0.77(0.87)	NR, p = .007
	sIL-6R pg/mL (serum)					37.33(21.08)/27.15(17.26)	NR, p < .05
Ibrahim and Afifi (2012)	IL-6 pg/mL (serum)	22(NR)/12(NR)	RRMS	31.2 (8.7)	BDI	8.9(3.2)/3.3(1.4)	NR, p < .001
	IL-6R pg/mL (serum)					39.58(24.12)/19.2(13.54)	NR, p < .001

*Notes*. Y = Yes, N = No Disease modifying medication. CI = Confidence Interval *M* = Mean, *SD* = Standard Deviation, OR = Odds Ratio, NR = not reported.

### 3.4 Changes in inflammatory protein concentrations after psychological treatment for depression in individuals with MS

Only one study assessed inflammatory protein concentrations in 14 participants with relapse remitting MS and depression before and after a cognitive behavioural therapy (CBT) intervention targeting depressive symptoms (Mohr et al., 2001) and found a significant reduction in IFN- γ concentration in serum after the CBT intervention compared to baseline.

### 3.5 Meta-analytic results of grouped inflammatory proteins

**Fig. 2:**
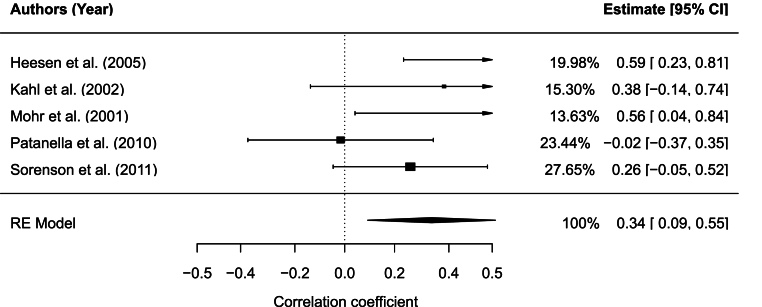
Forest plot for combined inflammatory protein meta-analysis.

Inflammatory protein measurements (TNF-a, IFN-γ, IFN-γ PHA, IL-10, IL-10 LPS, IL-10 PHA, IL-6, IL-6 LPS, IL-6 PHA) from serum βand analyzed using immunoassays were extracted from five studies and included a total of 125 MS subjects (Heesen et al., 2005; Kahl et al., 2002; Patanella et al., 2010; Sorenson et al., 2011; Mohr et al., 2001). When the study had more than one inflammatory protein measure, its average effect size and variance was calculated to treat the study as a single data point in the meta-analysis and avoid giving it extra weight due to multiple entries. The correlation between levels of depression and inflammatory proteins was significant, *r* = 0.35, 95% CI [0.09,0.61], *p* = .008 (see Fig. 2). The analysis indicated moderate, albeit nonsignificant, heterogeneity across studies, *Q*_(4)_ = 6.90, *p* = .141, *I*^2^ = 44.08%. There was no evidence of small study bias or publication bias (Egger's regression test: *p* = .240; Rank correlation test for funnel plot asymmetry: *p* *=* .483). No significant moderating effects of the presence of controls(Q_M_(4) = 6.90, *p* = .141), mean age(Q_M_(1) = 0.20, *p* = .652), medication(Q_M_(1) = 0.04, *p* = .833), study quality(Q_M_(1) = 0.03, *p* = .872), year of publication(Q_M_(1) = 2.93, *p* = .087), years since MS diagnosis(Q_M_(1) = 0.05, *p* = .825), type of depression measure used (BDI, BDI-II, HADS-D, MADRS-S, POMS-TD (Q_M_(1) = 1.15, *p* = .563) were found. Percent female showed a statistically significant moderating effect on the relationship between depression level and concentrate of inflammatory protein (Q_M_(1) = 5.31, *p* = .021).

### 3.6 Meta-analytic results of individual inflammatory proteins

Random effects meta-analysis models were performed on data for IFN- γ and IL-10 from serum that were analyzed in at least three studies (Heesen et al., 2005; Kahl et al., 2002; Patanella et al., 2010; Sorenson et al., 2011). We could not perform individual meta-analyses for TNF-α and IL-6 (LPS and PHA) due to the low amount of studies available. The moderating effects of the presence of controls, mean age, medication use, percentage female, study quality, year of publication, years since MS diagnosis, type of depression measure used and assay type were examined for each meta-analysis.

### 3.7 Studies of IFN- γ

**Fig. 3:**
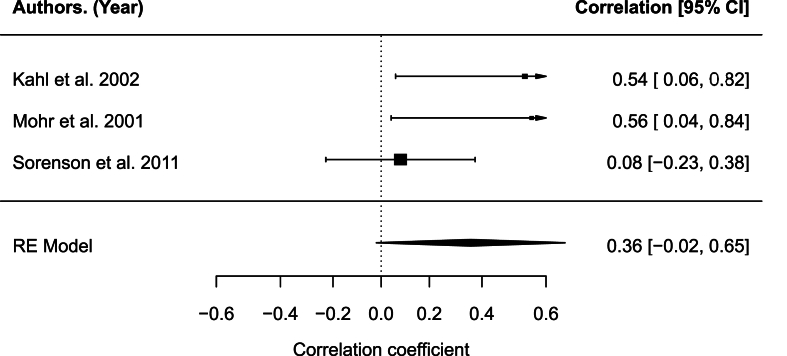
Forest plot for IFN-γ

Inflammatory protein measurements for IFN-γ from serum were extracted from three studies which used immunoassays and included 102 subjects (Kahl et al., 2002; Sorenson et al., 2011; Mohr et al., 2001). The correlation between level of depression and IFN-γ was not significant, *r* = 0.38, 95% CI [-0.02,0.77], *p* = .063 (see Fig. 3). The analysis indicated moderate heterogeneity across studies, *Q*_(2)_ = 4.78, *p* = .118, *I*^2^ = 52.83%. Although Egger's regression test was statistically significant (*p* = .039), this result should be interpreted with caution due to the small number of studies (*k* = 3), which limits the reliability of publication bias tests. The rank correlation test was not significant (p = .100), and there was no clear evidence of funnel plot asymmetry, suggesting that small-study effects or publication bias may not be a substantial concern. Year of publication (Q_M_(1) = 4.27, *p* = .039) and study quality (Q_M_(1) = 4.27, *p* = .039) showed a statistically significant moderating effect on the relationship between depression level and concentrate of IFN-γ cytokine. Other moderator variables were not significant: presence of controls(Q_M_(1) = 0.61, *p* = .434), mean age(Q_M_(1) = 0.56, *p* = .454), medication(Q_M_(1) = 0.39, *p* = .535), percentage female(Q_M_(1) = 0.24, *p* = .626, years since MS diagnosis(Q_M_(1) = 3.70, *p* = .054), type of depression measure used (BDI, POMS-TD (Q_M_(1) = 4.27, *p* = .039).

### 3.8 Studies of IL-10

**Fig. 4:**
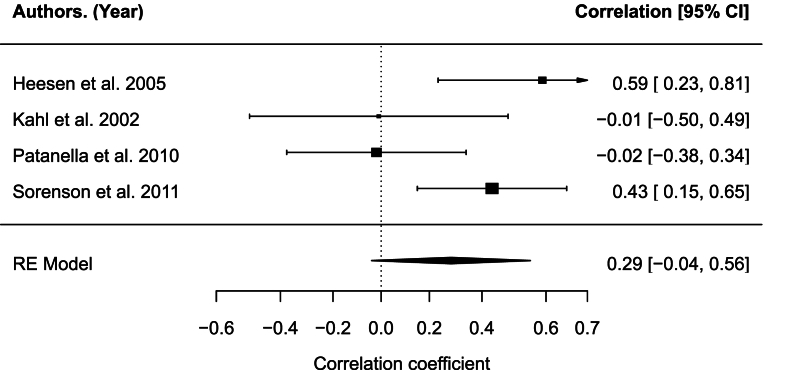
Forest plot for IL-10 LPS.

Cytokine measurements for IL-10 LPS and PHA from serum were extracted from four studies using immunoassays and included 111 subjects (Heesen et al., 2005; Kahl et al., 2002; Patanella et al., 2010; Sorenson et al., 2011). The correlation between levels of depression and IL-10 LPS was not significant, *r* = 0.29, 95% CI [-0.04,0.63], *p* = .085 (see Fig. 4). The analysis indicated moderate heterogeneity across studies, *Q*_(3)_ = 7.92, *p* = .048, *I*^2^ = 62.73%. There was no evidence of small-study effects or publication bias (Egger's regression test: p = .634; Rank correlation test: p = .100), with no significant funnel plot asymmetry. Type of depression measure (BDI, POMS-TD) showed a statistically significant moderating effect on the relationship between depression level and concentration of IL-10 LPS (Q_M_(2) = 7.92, *p* = .019), particularly for BDI (estimate = −0.69, *p* = .011) and not POMS-TD (estimate = −0.21, *p* = .442). The other candidate moderator variables were not significant: presence of controls(Q_M_(1) = 0.63, *p* = .427), mean age(Q_M_(1) = 2.00, *p* = .157), medication(Q_M_(1) = 0.31, *p* = .575), percentage female(Q_M_(1) = 2.23, *p* = .135), study quality(Q_M_(1) = 2.53, *p* = .112), year of publication(Q_M_(1) = 0.01, *p* = .912), years since MS diagnosis(Q_M_(1) = 0.06.

**Fig. 5:**
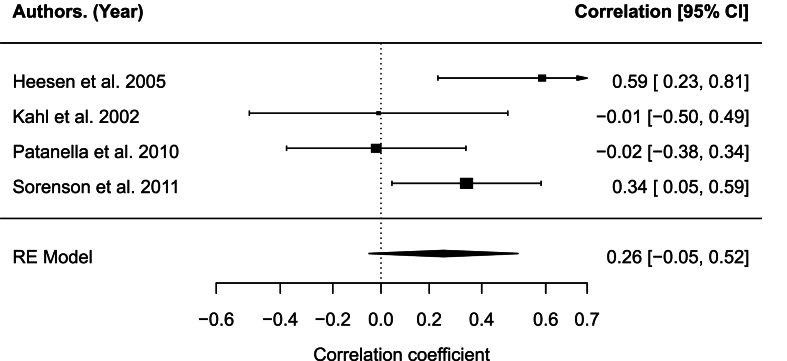
Forest plot for IL-10 PHA

The correlation between levels of depression and IL-10 PHA was not significant, r = 0.26, 95% CI [-0.05,0.58], p = .099 (see Fig. 5). The analysis indicated moderate heterogeneity across studies, Q(3) = 6.93, p = .074, I2 = 57.64%. There was no evidence of small-study effects or publication bias (Egger's regression test: p = .759; Rank correlation test: p = .100), with no significant funnel plot asymmetry. Type of depression measure (ADS-L, BDI, POMS-TD) showed a statistically significant moderating effect on the relationship between depression level and concentration of IL-10 PHA (Q_M_(2) = 6.93, *p* = .031), particularly for BDI (estimate = −0.69, *p* = .011) and not POMS-TD (estimate = −0.32, *p* = .248). The other candidate moderator variables were not significant: presence of controls(Q_M_(1) = 0.58, *p* = .447), mean age(Q_M_(1) = 1.09, *p* = .297), medication(Q_M_(1) = 0.10, *p* = .750), percentage female(Q_M_(1) = 3.47, *p* = .063, study quality(Q_M_(1) = 3.43, *p* = .064), year of publication(Q_M_(1) = 0.002, *p* = .967), and years since MS diagnosis(Q_M_(1) = 0.002, *p* = .967).

### 3.9 Methodological differences

The methodologies for handling and analyzing inflammatory proteins across the studies exhibited notable differences in assay types, assay kits, sensitivity and control measures. All studies in the meta-analyses used enzyme-linked immunosorbant assay (ELISA) kits from 6 different suppliers. Studies diverged in their manufacturer and kit type, resulting in variability in sensitivity and intra-inter-assay variation. Additionally, sensitivity of IFN- γ assays ranged from 1.5 pg/ml to 7 pg/mL, with intra- and interassay variations varying between 4.5% and 7.4%. Some studies employed control measures, such as the use of serum controls and performing assays in triplicate to monitor interassay reliability. Sample sources varied, with nine studies using serum (Rahimlou et al., 2020; Heesen et al., 2005, 2006; Kahl et al., 2002; Patanella et al., 2010; Rolf et al., 2017; Sorenson et al., 2011; Mohr et al., 2001; Vesic et al., 2018) and two using saliva samples and CSF (Rossi et al., 2017; Brenner et al., 2018). Nine studies used immunoassays (Rahimlou et al., 2020; Heesen et al., 2005, 2006; Kahl et al., 2002; Patanella et al., 2010; Rolf et al., 2017; Mohr et al., 2001; Vesic et al., 2018; Sorenson et al., 2011) and two studies used bioassays (Rossi et al., 2017; Brenner et al., 2018).

### 3.10 Assessment of data quality

Assessment of the quality of all cross-sectional studies was examined using an adapted version of the Newcastle-Ottowa scale with all studies falling in the ‘poor quality’ category (range 3–6) (see Supplementary Tables 5 and 6). Notably, ten studies fell short of quality assessment standards in the outcome and statistical categories. Specifically, included studies used self report scales and not independent blind assessments and clinical interviews to diagnose MDD. Most studies did not report confidence intervals around effect sizes. Only one intervention study was identified and assessed using the Newcastle-Ottowa Quality Assessment form for cohort studies which found that all categories were ‘low risk’ apart from selective reporting, other bias and incomplete outcome data categories, which contained insufficient information (Mohr et al., 2001).

## 4 Discussion

This is the first meta-analytic review to systematically synthesize the literature examining associations between level of depression and concentrations of inflammatory proteins. A grouped meta-analysis found significant positive associations between concentrations of TNF-a, IFN-γ, IFN-γ PHA, IL-10, IL-10 (LPS and PHA), IL-6, IL-6 (LPS and PHA) and levels of depression in individuals with MS. Where data was available, individual meta-analyses showed positive associations between IFN-γ and IL-10 and level of depression. Only one study examined changes in inflammatory proteins in adults with MS and depression after a CBT intervention and found reductions in IFN-γ cytokine concentration in those that undertook the CBT depression intervention (Mohr et al., 2001). These results are consistent with previous studies that found higher levels of TNF-a^20^, IFN-γ (Maes et al., 1994) IL-6 (Raison et al., 2006; Wiener et al., 2019), IL-10 levels (Wiener et al., 2019) in individuals with depression compared to healthy controls.

TNF-a has been implicated in the acute phase response where TNF-α acts like a hormone which is released by innate immune cells at the sites of infection or inflammation and released into circulation to act on distant organs like the liver and mediate the release of acute phase reactants like C-reactive protein (CRP). IFN-γ has been shown to also play a role in stress-induced immune dysregulation (Witek-Janusek et al., 2008) with elevated production of IFN-γ being observed in people with MS with fatigue and depression compared to those with MS and no fatigue (Pokryszko-Dragan et al., 2012). A meta-analysis (Dowlati et al., 2010) and recent study (Daria et al., 2020) found significantly decreased serum IFN-γ levels in people with major depressive disorder (MDD) compared to healthy controls. Although IFN-γ has been found to be decreased in base levels, IFN-γ was found to be significantly enhanced in patients with depression in response to mitogens (Maes et al., 1994).

IL-6 has been found to have pro- or anti-inflammatory properties. In reaction to stressful stimuli, the synthesis of IL-6 increases, and findings suggest that in depression, higher IL-6 levels correlate with a more severe course of the disease (Li et al., 2022). IL-6 has also been linked to specific symptoms or subtypes of depressive disorder such as reduced appetite, sleep disturbances, low mood, and feelings of worthlessness (Manfro et al., 2022). IL-6 was associated with depression in people experiencing the stressful events (Li et al., 2022). Similarly, IL-10 has been found to act as an anti-inflammatory cytokine (Saraiva et al., 2010; Yadav et al., 2021) with both decreased and increased levels of IL-10 found in people with depression. It has been suggested that IL-10 levels increase initially in response to acute inflammation as part of the immune system response connected with depression with IL-10 levels eventually decreasing over the course of depression (Wiener et al., 2019; Chi et al., 2021; Al-Fadhel et al., 2019; Roque et al., 2009).

Our results strengthen the evidence that depression in individuals with MS is accompanied by activation of the inflammatory response system and that a set of inflammatory proteins are involved in the chronic inflammatory processes that may underly the pathogenesis and pathophysiology of depression. The current results lend support to the cytokine hypothesis of depression which proposes that inflammatory cytokines and an immune system response are part of the development of MDD (Miller et al., 2002; Steptoe et al., 2003; Guidi et al., 1991; Heiser et al., 2008) and that illnesses characterized by chronic inflammatory responses, such as MS, are associated with increased rates of depression (Gold and Irwin, 2009).

## 5 Limitations

There are limitations to the current meta-analysis. The review was restricted to English language texts which may introduce language bias and exclude relevant studies published in other languages. Many clinical variables that may have affected the relationship between cytokines and depression were not examined or reported which may have affected our results. The moderating effects of methodological factors such as timing of blood sampling reflecting circadian variations (O'Connor et al., 2009), the menstrual cycle, use of disease modifying and psychoactive medications, level of ambulation and disability status, drug and alcohol use, oral health, fasting or non-fasting state of participants, assay reliability, the time interval between blood collection, separation and storage, the number of freeze-thaw cycles (Heiser et al., 2008) of the sample prior to analysis could not be investigated in the current meta-analysis due to lack of consistent information across all analyzed studies. Low study numbers for individual inflammatory proteins may also have confounded results.

Most studies controlled for age and gender in analyses although ethnicity, acute phase of MS and depressive illness, MS subtype, level of ambulation or disability, use of disease modifying and anti-depressant medications and comorbid medical diagnoses were not always reported or controlled for in analyses. Participants may have been taking anti-depressant medication although this information was not reported in some studies used in the meta-analyses except for three studies which excluded participants taking anti-depressant medication (Heesen et al., 2005; Patanella et al., 2010; Brenner et al., 2018; Mohr et al., 2001). All studies in the current review used a cut-off to determine whether individuals were classified as depressed or not, and cutoffs may have varied across the self-report depression scales.

Percentage of female participants was found to be a significant moderator in the meta-analysis where the inflammatory proteins were combined to examine their association with level of depression. For analyses related to IL-10 (PHA and LPS), type of depression measure was found to be a significant moderator. There was also moderate heterogeneity observed between studies which may be attributable to variability in assay procedures both within and between laboratories. However, type of assay was not found to be a significant moderator in the meta-analyses nor was there evidence of small study bias or publication bias for the inflammatory protein examined.

Additionally, most studies did not provide a justification for sample size used, or accounted for handling of non-responses or used independent blind assessment (i.e., clinical interview) to assess and diagnose depression. The majority of studies used convenience sampling and did not specify selection and recruitment of healthy control samples. Handling and analyzing inflammatory proteins across the different studies also varied with variability in the specific kits, sensitivities, and reporting across studies. Some studies employed control measures to monitor interassay reliability such as the use of serum controls and performing assays in triplicate. Causal inferences between inflammatory protein concentration and level of depression cannot be made due to the cross-sectional design of all studies included in this review.

Overall, studies were ranked as ‘poor quality’ due to limited outcome and statistical reporting of their studies. Only one intervention (Mohr et al., 2001) was identified and received an overall ‘fair quality’ rating but did include limitations such as use of self-report questionnaire to determine depression, limited description of the inflammatory protein collection procedure and the lack of outcome assessment procedure. Given the low quality of studies sensitivity analyses were not able to be conducted.

## 6 Future Directions

Future research examining level of depression and inflammatory protein concentrations in serum, saliva, cerebrospinal fluid and neuroimaging may assist in further extrapolating the relationship between TNF-α, IFN-γ, IL-6 and IL-10 and depression. Identifying a characteristic inflammatory protein profile in individuals with MS and depression, and examining how this profile differs in individuals with depression without MS and a healthy control group, will assist with assessment, diagnosis and treatment of depression. Future longitudinal research should focus on improving study quality and harmonizing data collection procedures including collecting and reporting information on potential moderators such as medication use, disease duration, MS subtype, and comorbid medical diagnoses in order to replicate the current findings and confirm the mechanisms through which inflammatory proteins are present in individuals with MS and depression. These are critical steps to advancing this field and addressing the inconsistencies across studies that this present review has identified.

## 7 Conclusion

With the data available, significant associations between TNF-a, IFN-γ, IL-6 and IL-10 concentrations and level of depression were found suggesting that depression is associated with inflammatory responses in the immune system in individuals with MS. However, the current results should be viewed as exploratory given the heterogeneity and small number of studies included in the current meta-analytic review.

## CRediT authorship contribution statement

**L.A. Kiropoulos:** Writing – review & editing, Writing – original draft, Validation, Supervision, Software, Resources, Project administration, Methodology, Investigation, Data curation, Conceptualization. **V. Rozenblat:** Writing – review & editing, Writing – original draft, Validation, Methodology, Formal analysis, Data curation, Conceptualization. **N. Baes:** Writing – review & editing, Writing – original draft, Validation, Methodology, Investigation, Formal analysis, Data curation.

## Declaration of generative AI and AI-assisted technologies in the writing process

No generative AI and AI-assisted technologies were used in the writing process of this manuscript.

## Declaration of competing interest

There are no conflict of interest among all authors.

## Data Availability

I have provided link to data
