# Supplementary material for: Inflammatory proteins related to depression in multiple sclerosis: A systematic review and meta-analysis
Source: Brain Behav Immun Health. 2024 Dec 28;43:100939. doi: 10.1016/j.bbih.2024.100939 (PMC11758135; doi:10.1016/j.bbih.2024.100939)

**Supplementary Table 1. Data checking for meta-analyses including funnel plots**

|  | **Data checking for meta-analyses** |
| --- | --- |
| **Transformation** | Prior to meta-analytic synthesis, correlation coefficients were transformed into the normally distributed Fischer’s *z* for variance stabilization^29^ and reverted to Pearson’s *r* for reporting the weighted average summary effect size.^30,31^ |
| **Outliers** | Outlier diagnostics were used to identify studies which may have disproportionately influenced heterogeneity.^32,33^ |
| **Small study bias** | Small study bias, which includes publication bias and study quality bias,^34,35^ was assessed by visually inspecting funnel plots alongside  Egger’s regression test for small study bias (*p* < .05). Publication bias was assessed using rank correlation tests (*p* < .05 indicates funnel plot asymmetry) and selection models which computed likelihood ratio tests to compare publication bias adjusted models (studies weighted by their p-values rather than sample sizes) and the original random effects models (*p* < .1). |
| **Heterogeneity** | Heterogeneity was assessed using Cochrane’s *Q*-statistic (which tests for variance in the true effect sizes between studies) and the *I^2^* statistic (which tests for heterogeneity across studies not due to random error): values of ~25%, ~50%, and ~75% interpreted respectively as low, moderate, high.^36^ We set the level of significance for Cochrane’s *Q* test to 0.10 due to its low power to detect heterogeneity.^36^ |

**Funnel plots**

**Combined meta-analysis**


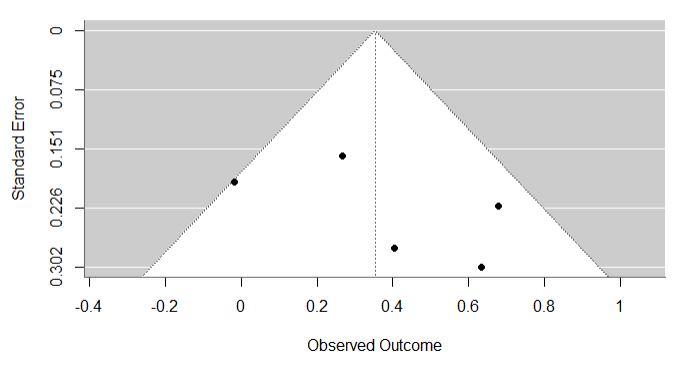


**Individual meta-analysis for IFN-g**


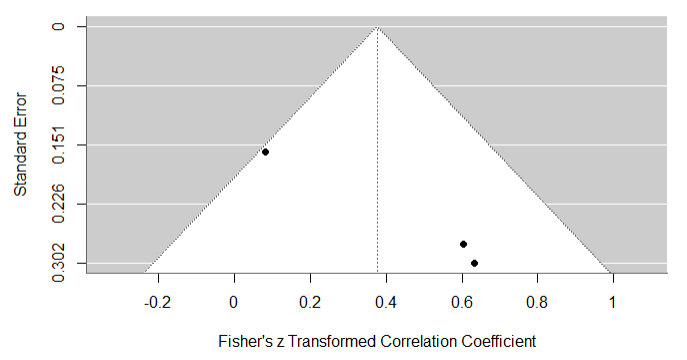


**Individual meta-analysis for IL-10 LPS**


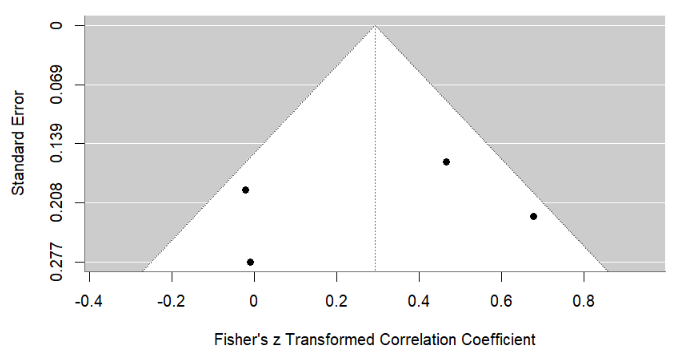


**Individual meta-analysis for IL-10 PHA**


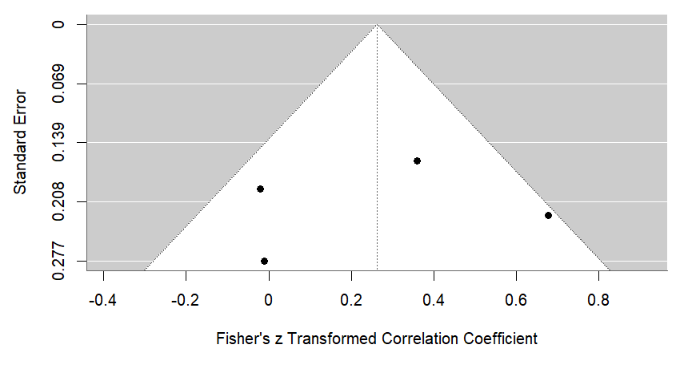

Supplement: Multimedia component 1 [file mmc1.docx]
