# Supplementary material for: Inflammatory proteins related to depression in multiple sclerosis: A systematic review and meta-analysis
Source: Brain Behav Immun Health. 2024 Dec 28;43:100939. doi: 10.1016/j.bbih.2024.100939 (PMC11758135; doi:10.1016/j.bbih.2024.100939)
Supplement: Multimedia component 2 [file mmc2.docx]

**Supplementary Table 2. Full text manuscripts excluded from review (n=52)**

| **Rationale for exclusion** | **Total** |
| --- | --- |
| No reported correlation between depression measure and cytokine concentration (1–15) | 15 |
| Conference abstracts/ insufficient information about the study (16–26) | 11 |
| No depression measure in MS sample (27–36) | 10 |
| No serum/plasma cytokine (i.e., cytokine level not provided) (37–40) | 4 |
| Data was not provided by researchers when requested (41–43) | 3 |
| Cytokine effect sizes were provided in under three papers overall (44,45) | 2 |
| Protocol paper (46,47) | 2 |
| No examination of cytokine in depressed and non-depressed (48) | 1 |
| Effect sizes for cytokine related analyses were unavailable (49) | 1 |
| Duplicate data (50) | 1 |
| Mixed depression-anxiety measure used (50-52) | 2 |

1. Berkovich R, Bakshi R, Amezcua L, Axtell RC, Cen SY, Tauhid S, et al. Adrenocorticotropic hormone *versus* methylprednisolone added to interferon β in patients with multiple sclerosis experiencing breakthrough disease: a randomized, rater-blinded trial. Ther Adv Neurol Disord. 2017 Jan;10(1):3–17.

2. Briken S, Rosenkranz SC, Keminer O, Patra S, Ketels G, Heesen C, et al. Effects of exercise on Irisin, BDNF and IL-6 serum levels in patients with progressive multiple sclerosis. J Neuroimmunol. 2016 Oct;299:53–8.

3. Browne RW, Jakimovski D, Ziliotto N, Kuhle J, Bernardi F, Weinstock-Guttman B, et al. High-density lipoprotein cholesterol is associated with multiple sclerosis fatigue: A fatigue-metabolism nexus? J Clin Lipidol. 2019 Jul;13(4):654-663.e1.

4. Ysrraelit MC, Gaitán MI, Lopez AS, Correale J. Impaired hypothalamic-pituitary-adrenal axis activity in patients with multiple sclerosis. Neurology. 2008 Dec 9;71(24):1948–54.

5. Moccia M, Capacchione A, Lanzillo R, Carbone F, Micillo T, Perna F, et al. Coenzyme Q10 supplementation reduces peripheral oxidative stress and inflammation in interferon-β1a-treated multiple sclerosis. Ther Adv Neurol Disord. 2019 Jan;12:175628641881907.

6. Loy BD, Fling BW, Sage KM, Spain RI, Horak FB. Serum histidine is lower in fatigued women with multiple sclerosis. Fatigue Biomed Health Behav. 2019 Apr 3;7(2):69–80.

7. Kierkegaard M, Lundberg IE, Olsson T, Johansson S, Ygberg S, Opava C, et al. High-intensity resistance training in multiple sclerosis — An exploratory study of effects on immune markers in blood and cerebrospinal fluid, and on mood, fatigue, health-related quality of life, muscle strength, walking and cognition. J Neurol Sci. 2016 Mar;362:251–7.

8. Tauil CB, da Rocha Lima AD, Ferrari BB, da Silva VAG, Moraes AS, da Silva FM, et al. Depression and anxiety in patients with multiple sclerosis treated with interferon-beta or fingolimod: Role of indoleamine 2,3-dioxygenase and pro-inflammatory cytokines. Brain Behav Immun - Health. 2020 Oct 23;9:100162.

9. Sánchez-López AL, Ortiz GG, Pacheco-Moises FP, Mireles-Ramírez MA, Bitzer-Quintero OK, Delgado-Lara DLC, et al. Efficacy of Melatonin on Serum Pro-inflammatory Cytokines and Oxidative Stress Markers in Relapsing Remitting Multiple Sclerosis. Arch Med Res. 2018 Aug;49(6):391–8.

10. Stürner KH, Stellmann JP, Dörr J, Paul F, Friede T, Schammler S, et al. A standardised frankincense extract reduces disease activity in relapsing-remitting multiple sclerosis (the SABA phase IIa trial). J Neurol Neurosurg Psychiatry. 2018 Apr;89(4):330–8.

11. Damasceno A, Moraes AS, Farias A, Damasceno BP, Dos Santos LMB, Cendes F. Disruption of melatonin circadian rhythm production is related to multiple sclerosis severity: A preliminary study. J Neurol Sci. 2015 Jun;353(1–2):166–8.

12. Durelli L, Bongioanni MR, Ferrero B, Ferri R, Imperiale D, Bradac GB, et al. Interferon alpha-2a treatment of relapsing-remitting multiple sclerosis: disease activity resumes after stopping treatment. Neurology. 1996 Jul;47(1):123–9.

13. Håkansson I, Johansson L, Dahle C, Vrethem M, Ernerudh J. Fatigue scores correlate with other self-assessment data, but not with clinical and biomarker parameters, in CIS and RRMS. Mult Scler Relat Disord. 2019 Nov 1;36:101424.

14. Hamamcioglu K, Reder A. Interferon-β regulates cytokines and BDNF: greater effect in relapsing than in progressive multiple sclerosis. Mult Scler J. 2007 May;13(4):459–70.

15. Heesen C, Schulz KH, Fiehler J, Von Der Mark U, Otte C, Jung R, et al. Correlates of cognitive dysfunction in multiple sclerosis. Brain Behav Immun. 2010 Oct;24(7):1148–55.

16. Bettencourt A, Leal B, Ferreira M, Carvalho C, Moreira I, Santos E, et al. Depression symptoms in multiple sclerosis patients – The role of IL1B. J Neurol Sci. 2017 Oct;381:242.

17. Maes M, Kallaur AP, Lopes J, Oliveira SR, Simão ANC, Ramon D, et al. Immune-inflammatory and oxidative and nitrosative stress (IO & NS) pathways in depression and multiple sclerosis (MS): Shared IO & NS pathways but less hyper-acute neuro-inflammation explain the increased incidence of depression in MS. Neurol Psychiatry Brain Res. 2016 Mar;22(1):16–7.

18. Melnikov MV, Hasaeva MA, Belousova OO, Murugin VV, Pashenkov MV, Boyко AN. The role of dopamine in regulating interactions of the immune and nervous system in multiple sclerosis. J Neurol Sci. 2015 Oct;357:e300.

19. Melnikov M, Boyko A, Pashenkov M. The influence of depression on Th17-immune response in multiple sclerosis. J Neurol Sci. 2017 Oct;381:653.

20. Ferreira AM, Leal B, Ferreira I, Brás S, Moreira I, Samões R, et al. Depression and anxiety in multiple sclerosis patients: The role of genetic variability of interleukin 1β. Mult Scler Relat Disord. 2021 Jul;52:102982.

21. Cathérine D, Annelien DP, Anne S, Luc A, Liesbeth VH, Gerlo S, et al. End of dose interval symptoms in patients treated with natalizumab: A role for serum cytokines? Mult Scler Relat Disord. 2020 Jun;41:102020.

22. Roh YS, Mowry EM, Fitzgerald KC. Initiation of Higher-Efficacy Disease-Modifying Therapy and Depressive Symptom Evolution in Patients with Multiple Sclerosis. In CMSC; 2019 [cited 2024 Feb 19]. Available from: https://cmsc.confex.com/cmsc/2019/meetingapp.cgi/Paper/6201

23. Imamura K, Suzumura A, Hayashi F, Marunouchi T. Cytokine production by peripheral blood monocytes/macrophages in multiple sclerosis patients. Acta Neurol Scand. 1993 Apr;87(4):281–5.

24. Tüzün E, Arsoy E, Akbayir E, Şen M, Mercan Ö, Gencer M, et al. Cognitive assessment and cytokine profile of multiple sclerosis patients presenting with only optic neuritis and myelitis. [cited 2024 Feb 19];25. Available from: https://avesis.istanbul.edu.tr/yayin/423b0fcb-cd31-4e2b-998e-e2e676203b3d/cognitive-assessment-and-cytokine-profile-of-multiple-sclerosis-patients-presenting-with-only-optic-neuritis-and-myelitis

25. Athanasopoulos D, Kitsos D, Protopapas N, Liverezas A, Karageorgiou C. Correlation of hormonal status and cytokine profiles with fatigue in patients with multiple sclerosis: a case-control study. In: InMULTIPLE SCLEROSIS JOURNAL. ). 1 OLIVERS YARD, 55 CITY ROAD, LONDON EC1Y 1SP, ENGLAND: SAGE PUBLICATIONS LTD; p. 178–178.

26. Howard JT, Wright BR, Blank S. Associations between systemic inflammation and cognitve impairment in a multiple sclerosis sample. Psychosom Med. 2012;74(3):A65.

27. Simpson S, Stewart N, Van Der Mei I, Otahal P, Charlesworth J, Ponsonby AL, et al. Stimulated PBMC-produced IFN- and TNF- are associated with altered relapse risk in multiple sclerosis: results from a prospective cohort study. J Neurol Neurosurg Psychiatry. 2015 Feb 1;86(2):200–7.

28. Rossi S, Motta C, Studer V, Rocchi C, Macchiarulo G, Barbieri F, et al. Interleukin-8 is associated with acute and persistent dysfunction after optic neuritis. Mult Scler J. 2014 Dec;20(14):1841–50.

29. Cannella B, Raine CS. The adhesion molecule and cytokine profile of multiple sclerosis lesions. Ann Neurol. 1995 Apr;37(4):424–35.

30. Carrieri PB, Provitera V, Rosa TD, Tartaglia G, Gorga F, Perrella O. Profile of Cerebrospinal Fluid and Serum Cytokines in Patients with Relapsing-Remitting Multiple Sclerosis. A Correlation with Clinical Activity. Immunopharmacol Immunotoxicol. 1998 Jan;20(3):373–82.

31. Trenova AG, Slavov GS, Draganova-Filipova MN, Mateva NG, Manova MG, Miteva LD, et al. Circulating levels of interleukin-17A, tumor necrosis factor-alpha, interleukin-18, interleukin-10, and cognitive performance of patients with relapsing-remitting multiple sclerosis. Neurol Res. 2018 Mar 4;40(3):153–9.

32. El-Tamawy M, Darwish M, Ahmed S, Abdelalim A, Moustafa EngyBS. Primary fatigue contributes to cognitive dysfunction in patients with multiple sclerosis. Egypt J Neurol Psychiatry Neurosurg. 2016;53(2):74.

33. Vollmer TL, Wynn DR, Alam MS, Valdes J. A phase 2, 24-week, randomized, placebo-controlled, double-blind study examining the efficacy and safety of an anti-interleukin-12 and -23 monoclonal antibody in patients with relapsing–remitting or secondary progressive multiple sclerosis. Mult Scler J. 2011 Feb;17(2):181–91.

34. Khoury SJ, Healy BC, Kivisäkk P, Viglietta V, Egorova S, Guttmann CRG, et al. A Randomized Controlled Double-Masked Trial of Albuterol Add-on Therapy in Patients With Multiple Sclerosis. ARCH NEUROL. 2010;67(9).

35. Muris AH, Smolders J, Rolf L, Thewissen M, Hupperts R, Damoiseaux J. Immune regulatory effects of high dose vitamin D3 supplementation in a randomized controlled trial in relapsing remitting multiple sclerosis patients receiving IFNβ; the SOLARIUM study. J Neuroimmunol. 2016 Nov;300:47–56.

36. Cleland BT, Papanek P, Ingraham BA, Harkins A, Garnier-Villarreal M, Woo D, et al. Determinants of low bone mineral density in people with multiple sclerosis: Role of physical activity. Mult Scler Relat Disord. 2020 Feb;38:101864.

37. Gold SM, Kruger S, Ziegler KJ, Krieger T, Schulz KH, Otte C, et al. Endocrine and immune substrates of depressive symptoms and fatigue in multiple sclerosis patients with comorbid major depression. J Neurol Neurosurg Psychiatry. 2011 Jul 1;82(7):814–8.

38. Melief J, De Wit SJ, Van Eden CG, Teunissen C, Hamann J, Uitdehaag BM, et al. HPA axis activity in multiple sclerosis correlates with disease severity, lesion type and gene expression in normal-appearing white matter. Acta Neuropathol (Berl). 2013 Aug;126(2):237–49.

39. Stampanoni Bassi M, Garofalo S, Marfia GA, Gilio L, Simonelli I, Finardi A, et al. Amyloid-β Homeostasis Bridges Inflammation, Synaptic Plasticity Deficits and Cognitive Dysfunction in Multiple Sclerosis. Front Mol Neurosci. 2017 Nov 21;10:390.

40. Bettencourt LBA, Ferreira A, Carvalho C, Moreira I, Costa PP, Da Silva B, et al. IL1B rs16944 and depression symptoms in multiple sclerosis patients. Eur J Neurol. 2017;24(1):445–678.

41. Ibrahim WSM, Afifi S. Value Of Interleukin-6 In Early Prediction Of Depression In Multiple Sclerosis. 2012;

42. Kallaur AP, Lopes J, Oliveira SR, Simão ANC, Reiche EMV, De Almeida ERD, et al. Immune-Inflammatory and Oxidative and Nitrosative Stress Biomarkers of Depression Symptoms in Subjects with Multiple Sclerosis: Increased Peripheral Inflammation but Less Acute Neuroinflammation. Mol Neurobiol. 2016 Oct;53(8):5191–202.

43. Koutsouraki E, Hatzifilipou E, Michmizos D, Cotsavasiloglou C, Costa V, Baloyannis S. Increase in Interleukin-6 Levels Is Related to Depressive Phenomena in the Acute (Relapsing) Phase of Multiple Sclerosis. J Neuropsychiatry Clin Neurosci. 2011 Oct;23(4):442–8.

44. Rolf L, Muris AH, Bol Y, Damoiseaux J, Smolders J, Hupperts R. Vitamin D 3 supplementation in multiple sclerosis: Symptoms and biomarkers of depression. J Neurol Sci. 2017 Jul;378:30–5.

45. Katarina V, Gordana T, Svetlana MD, Milica B. Oxidative stress and neuroinflammation should be both considered in the occurrence of fatigue and depression in multiple sclerosis. Acta Neurol Belg. 2020 Aug;120(4):853–61.

46. Joisten N, Rademacher A, Bloch W, Schenk A, Oberste M, Dalgas U, et al. Influence of different rehabilitative aerobic exercise programs on (anti-) inflammatory immune signalling, cognitive and functional capacity in persons with MS – study protocol of a randomized controlled trial. BMC Neurol. 2019 Dec;19(1):37.

47. Bahr LS, Bock M, Liebscher D, Bellmann-Strobl J, Franz L, Prüß A, et al. Ketogenic diet and fasting diet as Nutritional Approaches in Multiple Sclerosis (NAMS): protocol of a randomized controlled study. Trials. 2020 Jan 2;21(1):3.

48. Mikova O, Yakimova R, Bosmans E, Kenis G, Maes M. Increased serum tumor necrosis factor alpha concentrations in major depression and multiple sclerosis. Eur Neuropsychopharmacol. 2001 Jun;11(3):203–8.

49. Andlauer TFM, Grummel V, Gasperi C, Klein AK, Hoshi MM, Berthele A, et al. Characterization of cytokine profiles in multiple sclerosis. In: Multiple Sclerosis Journal [Internet]. Sweden; 2019 [cited 2021 Apr 5]. Available from: https://onlinelibrary.ectrims-congress.eu/ectrims/2019/stockholm/279188/till.andlauer.characterization.of.cytokine.profiles.in.multiple.sclerosis.html?f=menu%3D6%2Abrowseby%3D8%2Asortby%3D2%2Amedia%3D3%2Ace_id%3D1603%2Aot_id%3D21627

50. Mohr DC, Goodkin DE, Islar J, Hauser SL, Genain CP. Treatment of depression is associated with suppression of nonspecific and antigen-specific T(H)1 responses in multiple sclerosis. Arch Neurol. 2001 Jul;58(7):1081–6.

51. Heesen C, Nawrath L, Reich C, Bauer N, Schulz KH, Gold SM. Fatigue in multiple sclerosis: an example of cytokine mediated sickness behaviour?. *J Neurol Neurosurg Psychiatry*. 2006;77(1):34-39. doi:10.1136/jnnp.2005.065805

52. Kallaur AP, Lopes J, Oliveira SR, et al. Immune-Inflammatory and Oxidative and Nitrosative Stress Biomarkers of Depression Symptoms in Subjects with Multiple Sclerosis: Increased Peripheral Inflammation but Less Acute Neuroinflammation. *Mol Neurobiol*. 2016;53(8):5191-5202. doi:10.1007/s12035-015-9443-4
