# Supplementary material for: Inflammatory proteins related to depression in multiple sclerosis: A systematic review and meta-analysis
Source: Brain Behav Immun Health. 2024 Dec 28;43:100939. doi: 10.1016/j.bbih.2024.100939 (PMC11758135; doi:10.1016/j.bbih.2024.100939)
Supplement: Multimedia component 3 [file mmc3.docx]

**Supplementary Table 3: Characteristics of included studies examining differences in cytokine concentration in MS samples with versus without depression**

| **Study** | ***n* = MS + Dep** ***n* = MS – Dep** **(%female)** | ***n* = MS type** | **Disease modifying medication** | ***M* Age (yrs)** | ***M*(SD) yrs since MS dx** | **Depression measure & cut-off** | **Cytokine assay** | **Serum/**  **Saliva** | **Cytokine type & group *M*(SD)** | **Difference in cytokine level b/w dep and non-dep MS patients** |
| --- | --- | --- | --- | --- | --- | --- | --- | --- | --- | --- |
| Andlauer et al. (2019) | MS + Dep שּ  *n* = 25 (88%)  MS - Dep  *n* = 30 (77%)  Control ∞  *n* = 20 (75%) | RRMS  *n* = ‡  SPMS  *n* = ‡  PPMS  *n* = ‡  CIS  *n* = ‡ | Y | MS + Dep  31.8  MS – Dep  32.1  Control  30.2 | MS + Dep  ‡  MS – Dep  ‡  Control  N/A | BDI-II  none = < 10  mild = 10-18  moderate = 19-29  severe = > 29 | Immunoassay and Bioassay | CSF | **CCL4 – CSF**  MS + Dep = ‡  MS - Dep = ‡  Control = ‡  **CCL4 – serum**  MS + Dep = ‡  MS - Dep = ‡  Control = ‡  **CRP – CSF**  MS + Dep = ‡  MS - Dep = ‡  Control = ‡  **CRP – serum**  MS + Dep =  MS - Dep = ‡  Control = ‡  **FGF2 – CSF**  MS + Dep = ‡  MS - Dep = ‡  Control = ‡  **FGF2 – serum**  MS + Dep = ‡  MS - Dep = ‡  Control = ‡  **IL-9 – CSF**  MS + Dep = ‡  MS - Dep = ‡  Control = ‡  **IL-9 – serum**  MS + Dep = ‡  MS - Dep = ‡  Control = ‡  **TNF-α – CSF**  MS + Dep = ‡  MS - Dep = ‡  Control = ‡  **TNF-α – serum**  MS + Dep = ‡  MS - Dep = ‡  Control = ‡ | **CCL4 – CSF**  OR = ≥ 1.53;  *p* < .05  **CCL4 – serum**  OR ≥ 1.53; *p* < .05  **CRP – CSF**  OR = ≥ 1.53; *p* < .05  **CRP – serum**  OR = ≥ 1.53; *p* < .05  **FGF2 – CSF**  OR = ≥ 1.53;  *p* < .05  **FGF2 – serum**  OR = ≥ 1.53;  *p* < .05  **IL-9 – CSF**  OR = ≥ 1.53;  *p* < .05  **IL-9 – serum**  OR = 2.29; *p* < .05  **TNF-α – CSF**  OR = 1.17; *p* < .05  **TNF-α – serum**  OR = 2.07; *p* < .05 |
| Kallaur et al. (2016) | MS + Dep  *n* = 42 (83%)  MS - Dep  *n* = 108 (70%)  Control ∞  *n* = 249 (71%) | RRMS  *n* = ‡  SPMS  *n* = ‡  PPMS  *n* = ‡  CIS  *n* = ‡ | Y | MS + Dep  44.8 (14.4)  MS – Dep  40.1 (13.5)  Control  36.7 (10.9) | MS + Dep  ‡  MS – Dep  ‡  Control  N/A | HADS  > 8 | Immunoassay | Serum | **CRP mg/L** ▪  MS + Dep = 3.24 (4.31)  MS – Dep = 2.53 (3.54)  Control = 2.95 (4.72)  **IL-1 ng/mL** ▪  MS + Dep = 4.0 (4.2)  MS - Dep = 5.0 (6.8)  Control = 9.2 (29.3)  **IL-4 ng/mL** ▪  MS + Dep = 3.2 (5.6)  MS - Dep = 4.6 (7.1)  Control = 1.4 (1.5)  **IL-6 ng/mL** ▪  MS + Dep = 10.7 (32.7)  MS - Dep = 4.2 (6.3)  Control = 5.3 (14.7)  **IL-10 ng/mL** ▪  MS + Dep = 5.9 (3.2)  MS - Dep = 5.1 (3.3)  Control = 6.8 (8.8) | **CRP**  ‡  **IL-1**  ‡  **IL-4**  OR = .53*, CI = .28, .98  *p* = .044  **IL-6**  OR = 2.43**, CI = 1.36, 4.35  *p* = .003  **IL-10**  ‡ |
| Koutsouraki et al. (2011) | MS Acute + Dep  *n* = 13 ‡  MS Acute – Dep  *n* = 15 ‡  Control ∞  *n* = 20 ‡  MS Remission  *n* = 14 ‡ | RRMS  *n* = ‡  SPMS  *n* = ‡ | N | MS Acute + Dep  34.62 (8.16)  MS Acute – Dep  38.2 (8.75)  Control  ‡  MS Remission  37.71 (9.36) | MS Acute + Dep  ‡  MS Acute – D ep  ‡  Control  N/A  MS Remission  ‡ | BDI  > 15 | Immunoassay | Serum | **IL-6 pg/mL**  MS Acute + Dep  6.04 (8.80)  MS Acute - Dep  0.77 (0.87)  Control  ‡  MS Remission  1.17 (1.11)  **sIL-6R pg/mL**  MS Acute + Dep  37,338 (21,008)  MS Acute - Dep  27,158 (17,267)  Control  ‡  MS Remission  18,407 (4,914) | **IL-6**  MS Acute  *p* = .007  MS Remission  ‡  **sIL-6R**  MS Acute  *p* < .05  MS Remission  ‡ |
| Ibrahim & Afifi (2012) | MS Acute  *Total n* = 34 (65%)  MS Acute + Dep  *n* = 22 ‡  MS Acute – Dep  *n* = 12 ‡  Control ∞  *n* = 20 (60%)  Remission  *n* = 18 (72%) | Total RRMS  *n* = 52  MS Acute  *n* = 34  Remission *n* = 18 | Y | MS Acute  31.2 (8.7)  MS Acute + Dep  ‡  MS Acute – Dep  ‡  Control ∞  31.7 (5.1)  Remission  30.1 (9.3) | MS Acute  0.53(0.19)  MS Acute + Dep  ‡  MS Acute – Dep  ‡  Control ∞  N/A  Remission  0.43(0.09) | BDI  > 15 | Immunoassay | Serum | **IL-6 pg/mL**  MS + Dep = 8.9 (3.2)  MS – Dep = 3.3 (1.4)  **IL-6R pg/mL**  MS + Dep = 39.587 (24.124)  MS – Dep = 19.284 (13.541) | **IL-6**  MS Acute  *p* < .001  Remission  ‡  **IL-6R**  MS Acute *p* < .001  Remission  ‡ |

*Notes*. Y = Yes, N = No Disease modifying medication. HADS = Hospital Anxiety and Depression Scale. CI = Confidence Interval *M*= Mean, *SD*= Standard Deviation, Dep = Depression, ^שּ^Major Depressive Disorder, dx = Diagnosis, OR = Odds Ratio, * = *p* <.05, ** = *p* <.001, ‡ = not specified. ∞ = Control samples comprise non-MS and non-Depressed healthy controls, some samples age and gender matched. ‡~ = Three-way comparison included MS + dep, MS – Dep and a healthy-control group. ꚜ = data processed in Ln (natural log) transformation. CMS = Conventional MS. SCON = Spinal cord and optic nerve attacks. NS = non-significant p-value.
