# Supplementary material for: Inflammatory proteins related to depression in multiple sclerosis: A systematic review and meta-analysis
Source: Brain Behav Immun Health. 2024 Dec 28;43:100939. doi: 10.1016/j.bbih.2024.100939 (PMC11758135; doi:10.1016/j.bbih.2024.100939)
Supplement: Multimedia component 4 [file mmc4.docx]

**Supplementary Table 4: Study characteristics and associations between cytokine concentrations and level of depression in individuals with MS**

| **Study** | ***N = MS* (%female)**  ***N = Controls***∞ | **MS Type = n** | **Disease modifying medication** | ***M* age (SD)** | ***M*(SD) [range] yrs since MS dx** | **Cytokine assay** | **Serum/**  **Saliva** | **Cytokine type & group *M (SD)* [range] pg/mL** | **Depression measure & group *M* (SD) [range]** | **Correlation between depression and biomarker level in MS sample** |
| --- | --- | --- | --- | --- | --- | --- | --- | --- | --- | --- |
| Brenner et al. (2018) | *N* = 47 (31%)  *N* = N/A | RRMS  *n* = 47 | Y | MS  34^ | 0^ [0-11] | Bioassay | CSF | **IL-6**  3.5^ [0.7, 65]  **IL-8**  47^ [16, 90] | MADRS-S  7^ [0, 29] | **IL-6**  *r* = 0.41  *p* < .01  **IL-8**  *r* = -0.09  *p* > .05 |
| Heesen et al. (2005) | *N* = 23 (78%)  *N* = 25 (80%)  ∞ | RRMS  *n* = 19  SPMS  *n* = 3  RPMS  *n* = 1 | Y = 14  N = 9 | MS  40.13 (2.23)  Control  37.80 (1.97) | 10.23 (2.04)  Control  N/A | Immunoassay | Serum | **IFN-γ**  ‡  **IL-10**  ‡  **TNF-α**  ‡ | ADS-L  MS: 19.1 (9.2)  Control: 5.64 (3.6) | **IFN-γ**  ‡  **IL-10**  *r* = 0.59  *p* = .003  **TNF-α**  ‡ |
| Heesen et al. (2006) | *N* = 30 (60%)  *N* = N/A | RRMS  *n* = 17  SPMS  *n* = 10  PPMS  *n* = 3 | Y = 5  N = 25 | MS + fatigue  46.6 (11.69)  MS – fatigue  42.87 (10.17) | MS + fatigue  13.80 (9.68)  MS – fatigue  8.60 (5.57) | Immunoassay | Serum | **IFN-γ**  MS + fatigue  57.6 (41.6)  MS – fatigue  27.8 (37.1)  **IL-10**  MS + fatigue  128.2 (47.0)  MS - fatigue  97.0 (52.3)  **TNF-α**  MS + fatigue  478.9 (209.7)  MS – fatigue  228.2 (208.1) | HADS  MS + fatigue  7.93 (3.30)  MS - fatigue  3.13 (3.50) | **IFN-γ**  *r* = 0.08  *p* = .67  **IL-10**  *r* = -0.001  *p* = .99  **TNF-α**  *r* = 0.08  *p* = .70 |
| Kahl et al. (2002) | *N* = 16 (75%)  *N* = 10 (80%)  ∞ | RRMS = 16 | Y | MS  30.1 (5.8)  Control  31 ‡ | MS  ‡  Control  N/A | Immunoassay | Serum | **IFN-γ**  ‡  **IL-4**  ‡  **IL-10**  ‡  **TNF-α**  ‡ | BDI  MS ¥  13.6 (5.5)  Control ¥  4.6 (2.5) | **IFN-γ** ¥  *r* = .54  *p* = .03  **IL-4** ¥  *r* = .13 *p* = .96  **IL-10** ¥  *r* = -.01  *p* = .97  **TNF-α** ¥  *r* = .55  *p* = .03 |
| Mohr et al. (2001) | *N* = 14 (71%)  *N* = 8 (62 %)  ∞ | RRMS  *n* = 14 | N | MS  47.4 [29-69]  Control  31.2 [23-41] | MS  11.3 [0.83 – 19.80]  Control  N/A | Immunoassay | Serum | **IFN-γ** ▪¥  Treatment  Baseline: 1177(939)  Week8: 702(416)  Post week16: 575(396)  Control  Baseline: 808(394)  Week8: 869(490)  Post week 16: 736(387)  **IFN-γ + IL-10** ▪¥▪  Treatment  Baseline: 537(484)  Wk8: 249(224)  Post week 16: 186(204)  Control  Baseline: 2.8(6.0)  Week8: 0(0)  Post week 16: 0.4(1.1)  **IL-4** ▪¥  Treatment  Baseline: 56(44)  Wk8: 28(26)  Post week 16: 44(57)  Control  Baseline: 12.8(12.0)  Week8: 7.9(8.7)  Post week 16: 5.6(6.6) | BDI  MS  Baseline: 21.9 (6.35)  Wk8: 15.6(4.9)  PostT: 14.2(9.1)  Control  Baseline: 3.6(3.0)  Week8: 2.5(3.1)  PostT: 4.1(3.8) | **IFN-y** ▪¥  *r* = 0.56  *p* = .04  **IFN- γ + IL-10** ▪¥▪  *r* = NS‡  *p* = .74  **IL-4** ▪¥  *r* = NS‡  *p* ≥ .22 |
| Patanella et al. (2010) | *N* = 30 (57 %)  *N* = 30 (57 %)  ∞ | RRMS  n = 30 | Y | MS  34.6 (5.7)  Control  34.6 (5.3) | 8 (1.75) | Immunoassay | Serum | **BDNF**  MS  876.9 (753.6)  Control  2318.5 (1513.3)  **IL-6**  MS  680.3 (1032.4)  Control  725.4 (885.7)  **IL-10**  MS  197.7 (103.3)  Control  82.4 (12.7)  **TNF-α**  MS  57.7 (69.3)  Control  36.9 (13.3) | BDI  MS  ‡  Control  ‡ | **BDNF**  *r =* -0.10  *p =* .58  **IL-6**  *r =* -0.12  *p =* .54  **IL-10**  *r =* -0.02  *p =* .90  **TNF-α**  *r =* 0.09  *p =* .65 |
| Rahimlou et al. (2020) | N = 65 (72%) | RRMS  n = 65 | N | Intervention  42.15 (11.98)  Control  39.9 (8.76) | Intervention  7.75 (3.99)  Control  5.66 (2.53) | Immunoassay | Serum | **BDNF ng/mL**  Intervention  Baseline: 14.92 (4.78)  Post-6-months: 17.19 (3.87)  Control  Baseline: 16.62 (6.49)  End of study: 15.71 (6.36)  **IL-6 pg/dL**  Intervention  Baseline: 11.99 (6.32)  Post-6-months: 5.28 (3.65)  Control  Baseline: 9.16 (4.51)  Post-6-months: 8.51 (4.70)  **NGF pg/mL**  Intervention  Baseline: 374.37 (76.42)  Post-6-months: 368.75 (78.75)  Control  Baseline: 370.47 (106.16)  Post-6-months: 371.04 (98.17) | BDI-II  Intervention  Baseline: 22.15 (1.62)  End of study: 17.34 (1.74)  Control  Baseline: 20.84 (1.25)  End of study: 18.93 (1.39) | **BDNF**  Intervention●:  *r* = 0.36  *p* = .042  Control○:  *r* = 0.24  *p* = .18  **IL-6**  Intervention●:  *r* = 0.005  *p* = .98  Control○:  *r* = 0.−17  *p* = .33  **NGF**  Intervention●:  *r* = 0.22  *p* = .23  Control○:  *r* = 0.05  *p* = .77 |
| Rolf et al. (2017) | *N* = 40 (65%) | RRMS  *n* = 40 | Y | Treatment  38.5 (7.8)  Placebo  37.6 (9.6) | Treatment  7.5^ ‡  Placebo  5.7^ ‡ | Immunoassay | Serum | **IL-10**  Placebo  Baseline: 1306^ {919–1754}  Post week48: 1762^ {1117–2496}  Treatment:  Baseline: 1100^ {673–2125}  Post week48: 1543^ {1021–2104}  **TNF-α**  Placebo  Baseline: 1035^ {331–1701}  Post week48: 765^ {302–1535}  Treatment:  Baseline: 879^ {393–1513}  Post week48: 932^ {648–1662} | HADS-D  Placebo  Baseline: 3.0^ [2.0–7.0]  Post week48: 2.0^ [1.0-6.5]  Treatment  Baseline:4.0^ [2.0–5.0]  Post week48: 3.0^ [2.0-5.0] | **IL-10**  *r* = NS‡  *p* > .05  **IFN-y**  *r* = NS‡  *p* > .05  **TNF-α**  *r* = NS‡  *p* > .05 |
| Rossi et al. (2017) | *N* = 405 ‡ | Total RRMS  *n* = 405  MS Acute  *n* = 54  Remission  *n = 57* | Y | MS  ‡  Control  ‡ | MS  ‡  Control  ‡ | Bioassay | CSF | **IL-1β**  ‡  **IL-2**  ‡  **IL-8**  ‡  **TNF- α**  ‡ | BDI-II  Baseline  13.94 (11.0)  3-month follow-up  9.13 (9.49) | **IL-1β**  *r* = 0.52  *p* < .001  **IL-2**  *r* = -0.06  *p* = .52  **IL-8**  *r* = 0.11  *p* = .22  **TNF-α**  *r* = 0.50  *p* < .001 |
| Sorenson et al. (2011) | *N* = 42 (71.4%)  *N* = 36 (8%)  ∞ | RRMS  *n* = 24  Chronic Progressive  *n* = 6  Progressive recurring  *n* = 1  Unidentifiable  *n* = 9 | MS  Y=1  N=41  Control  N/A | MS  49 (9.11), [28-75]  Control  43 (1.34), [29-54] | MS  49 (9.11)  Control  ‡ | Immunoassay | Serum | **IFN-γ**  ‡  **IL-6**  ‡  **IL-10**  ‡  **IL-12**  ‡  **TNF-α**  ‡ | POMS-TD  MS  ‡  Control  ‡ | **IFN-γ PHA*******  *r* = 0.081  *p* > .05  **IL6-LPS***  *r* = 0.02  *p* > .05  **IL6-PHA****  *r* = 0.386  *p* < .05  **IL-10 LPS*****  *r* = 0.435  *p* < .01  **IL-10 PHA******  *r* = 0.345  *p* < .05 |
| Vesic et al. (2018) | *N* = 98 (66%)  *N* = 35 ‡ ∞ | 14 | N | RRMS  41.7 (9.13)  MS acute 38.6 (7.88)  Control  ‡ | RRMS  6.19 (7.11)  MS acute 8.64 (4.46) | Immunoassay | Serum | **CRP ◊**  RRMS  1.01 mg/L (0.38)  MS acute  4.46 mg/L (0.40) | BDI  MS relapse  15.68 (16.62)  MS acute  8.36 (7.10)  Control  ‡ | **CRP ◊**  *r* = 0.40  *p* < .001 |

**Table 2 notes**

*Notes*. Y = Yes, N = No Disease modifying medication. ^ = Median value. *M*= Mean. *SD*= Standard Deviation. ADS-L = Allgemeine Depressions-Skala Lang. MADRS-S = Montgomery-Asberg Depression Rating Scale, self-report. *r*= correlation. ‡ = not specified. ∞ = Control samples comprise non-MS healthy controls, some samples age and gender matched. ᶺ¥ = OKT3 stimulated cytokine. ᶺ¥ᶺ = IL-10 suppression of OKT3 stimulated IFN-y. ¥ = levels measured during MS acute phase. * = *n=36; ** =* *n=37; *** = n=39; *****= *n=39; ******= *n=35*. LPS = lipopolysaccharide. PHA = phytohemagglutinin. {} = Q1-Q3. ꚜ = probiotic intervention. ꭛ = placebo. ꙴ  = metrics given in mg/L.
