# Supplementary material for: Inflammatory proteins related to depression in multiple sclerosis: A systematic review and meta-analysis
Source: Brain Behav Immun Health. 2024 Dec 28;43:100939. doi: 10.1016/j.bbih.2024.100939 (PMC11758135; doi:10.1016/j.bbih.2024.100939)
Supplement: Multimedia component 5 [file mmc5.docx]

## **Supplementary Table 5. Quality assessment of studies based upon adapted Newcastle-Ottawa scale (in Supplementary Materials)**

| **Author** |  | **Selection** | | | | | |  | **Comparability** | |  | **Outcome** | |  | **Statistical Test** | |  | **Total** | |
| --- | --- | --- | --- | --- | --- | --- | --- | --- | --- | --- | --- | --- | --- | --- | --- | --- | --- | --- | --- |
|  |  | 1 | | 2 | 3 | 4 | 5 |  | 1 | |  | 1 | |  | 1 | |  |  |  |
|  |  | a | b |  |  |  |  |  | a | b |  | a | b |  |  |  |  |  |  |
| Andlauer et al. (2019) |  | – | * | – | * | – | * |  | * | * |  | – | – |  | – |  |  | 5 |  |
| Kallaur (2016) |  | – | * | – | * | – | * |  | * | * |  | – | * |  | * |  |  | 7 |  |
| Koutsouraki et al. (2011) |  | – | * | – | * | – | * |  | * | * |  | – | – |  | – |  |  | 5 |  |
| Ibrahim & Afifi (2012) |  | – | * | – | * | – | * |  | – | – |  | – | – |  | – |  |  | 3 |  |
| Brenner et al. (2018) |  | – | * | – | * | – | * |  | * | * |  | – | – |  | – |  |  | 5 |  |
| Heesen et al. (2005) |  | – | * | – | * | – | * |  | – | – |  | – | – |  | – |  |  | 3 |  |
| Kahl (2002) |  | – | * | – | * | – | * |  | – | – |  | – | – |  | – |  |  | 3 |  |
| Patanella et al. (2010) |  | – | * | – | * | – | * |  | * | * |  | – | – |  | – |  |  | 5 |  |
| Rolf et al. (2017) |  | * | – | – | – | * | * |  | – | – |  | – | – |  | – |  |  | 3 |  |
| Rossi et al. (2017) |  | – | * | – | * | – | * |  | * | – |  | – | – |  | – |  |  | 4 |  |
| Sorenson et al. (2011) |  | – | * | – | * | – | * |  | * | – |  | – | – |  | – |  |  | 4 |  |
| Vesic et al. (2018) |  | – | * | – | * | – | * |  | * | * |  | – | – |  | – |  |  | 5 |  |

According to the standards of the adapted Newcastle-Ottawa scale, all cross-sectional studies fell in the "poor quality" category for AHRQ standards. Notably, most studies fell short of quality assessment standards in the outcome and statistical assessment categories. Specifically, independent blind assessments (i.e., clinical interviews) were not used to screen for depression; most were self-reports or no description. Additionally, most studies did not report in full their statistical results (e.g., confidence intervals were not reported around effect sizes). It should be noted that studies would have rated as “good quality” without including the outcome and statistical category.

## **Supplementary Table 6. Quality assessment of Mohr (2001) based on the Newcastle-Ottawa Quality Assessment Form for Cohort Studies**

| **Author** |  | **Selection** | | | | |  | **Comparability** | |  | **Outcome** | |  |  |  |  | **Total** | |
| --- | --- | --- | --- | --- | --- | --- | --- | --- | --- | --- | --- | --- | --- | --- | --- | --- | --- | --- |
|  |  | 1 | | 2 | 3 | 4 |  | 1 | |  | 1 | | 2 | 3 |  |  |  |  |
|  |  | a | b |  |  |  |  | a | b |  | a | b |  | a | b |  |  |  |
| Mohr (2001) |  | * | – | * | – | – |  | * | * |  | – | – | * | * | – |  | 6 |  |

Furthermore, Mohr's (2001) cohort study received a "fair quality" rating according to the Newcastle-Ottawa scale AHRQ standards.

*This section refers to Table 5 in the excel file “quality_appraisal_table.xlsx” which uses the Cochrane Risk of Bias Tool for the RCT:*

Because Rahimlou et al. (2019) employed a randomised control design, the Cochrane Risk of Bias Tool was used to rate its quality. All of its categories were judged to be “low risk” apart from Selective Reporting, Other Bias, and Incomplete Outcome Data. For these categories, the study contained insufficient information to permit judgement of ‘Low risk’ or ‘High risk’. Particularly, it was difficult to judge presence of incomplete outcome data due to a lack of evidence of any pre-registration of hypotheses and methods. Otherwise, there was low risk of bias during the procedure for randomizing participants as randomization lists were computer-generated by a statistician before being passed to the interviewer and researchers, patients, neurologists, and staff were blinded to the intervention and the placebo intervention. Furthermore, while it is difficult to blind the outcome assessment when using patient questionnaires, these instruments are developed to account for this and, because double blinding otherwise took place, this category was judged as low risk of bias.
